# Pegging Away

**Published:** 1892-02-06

**Authors:** 


### PASSING TOPICS.

### PEGGING AWAY.

Hospital secretaries are in no danger of lapsing into even momentary forgetfulness of the superlative merit of perseverance. In these days of keen competition everywhere, workers cannot afford to nod if they would ensure anything like success; attention must never be relaxed, and energy must not be allowed to languish. There are few people who more than charity officials are rendered conscious of the truth of this by everyday experience. We know that pushing traders depend largely upon the cleverness of their advertisements, and endeavour with more or less pertinacity to raise their announcements to the level of high art. But is it so very difficult to dispose of goods as tangible as soap, blacking, biscuits, patent medicines, and what not? We believe, and many illustrations in point could be given, that in regard to such commodities it is only necessary to advertise often enough and with sufficient boldness to ensure a rush of purchasers. The case is quite different with those who have nothing to offer to the classes appealed to but a tribute of gratitude and the consciousness of having helped forward a work of humanity. Advertisers of this description are sorely puzzled at times with the problem how to gain the world's attention, and to such we commend a careful study of the very clever appeals put forward on behalf of the various undertakings comprehended in the far-reaching efforts of the so-called "Church Extension Association." For originality, persistence, and a certain mingling of quaint humour and pathos, we know nothing in the nature of an appeal which compares with the emendations published in "Our Work."

Of course, it might be objected that appeals of the kind are deficient in dignity. But the real question is whether they are effective for the purpose in view. If the question of dignity were discussed in the abstract, many of us might easily arrive at the conclusion that "pegging away" at an unwilling public is not the most becoming method of maintenance for an established institution; but we must not overlook the fact that if workers generally took this view the tide of the world's practical benevolence would soon be at so low an ebb that many a useful venture would make shipwreck. We, who have seen plenty of appeals in our day, some of them successful and some otherwise, had no idea there was so much to be learned in the art. Yet not being lost to all sense of fitness, we will not at present recommend hospitals to appeal for gifts in kind, though we feel persuaded that if they did they would obtain many an offering from people who would never be persuaded to part with cash. This is not mere speculation. We recall the case of a certain old lady who was firmly convinced that hospitals were evils—"Necessary evils, I grant you," she would say, "but still evils"; yet who so far relented before expiring as to bequeath a whole shopful of harmoniums to several favoured institutions. If all else fails, we may see reason to return to this notion; but just now we have no difficulty in perceiving that our hospitals, like present-day brides, prefer the simplicity of a cash gift to the possession of multitudinous articles of more or less doubtful utility.

